# Lymphovascular invasion is a significant risk factor for non-sentinel nodal metastasis in breast cancer patients with sentinel lymph node (SLN)-positive breast cancer: a cross-sectional study

**DOI:** 10.1186/s12957-023-03273-6

**Published:** 2023-12-14

**Authors:** Chunyu Wei, Yongqing Deng, Suosu Wei, Zhen Huang, Yujie Xie, Jinan Xu, Lingguang Dong, Quanqing Zou, Jianrong Yang

**Affiliations:** 1 Department of Breast and Thyroid Surgery, People’s Hospital of Guangxi Zhuang Autonomous Region, Nanning, Guangxi China; 2 The Family Planning Office of the People’s Hospital of Guangxi Zhuang Autonomous Region, Nanning, Guangxi China; 3 Department of Scientific Cooperation of Guangxi Academy of Medical Sciences, People’s Hospital of Guangxi Zhuang Autonomous Region, Nanning, Guangxi China

**Keywords:** Breast cancer, Lymph nodes metastases, Lymphovascular invasion, Axillary lymph node dissection

## Abstract

**Background:**

A connection between lymphovascular invasion and axillary lymph node metastases in breast cancer has been observed, but the findings are inconsistent and primarily based on research in Western populations. We investigated the association between lymphovascular invasion and non-sentinel lymph node (non-SLN) metastasis in breast cancer patients with sentinel lymph node (SLN) metastasis in western China.

**Methods:**

This study comprised 280 breast cancer patients who tested positive for SLN through biopsy and subsequently underwent axillary lymph node dissection (ALND) at The People’s Hospital of Guangxi Zhuang Autonomous Region between March 2013 and July 2022. We used multivariate logistic regression analyses to assess the association between clinicopathological characteristics and non-SLN metastasis. Additionally, we conducted further stratified analysis. Results: Among the 280 patients with positive SLN, only 126 (45%) exhibited non-SLN metastasis. Multivariate logistic regression demonstrated that lymphovascular invasion was an independent risk factor for non-SLN in breast cancer patients with SLN metastasis (OR = 6.11; 95% CI, 3.62–10.32, *p* < 0.05). The stratified analysis yielded similar results.

**Conclusions:**

In individuals with invasive breast cancer and 1–2 positive sentinel lymph nodes, lymphovascular invasion is the sole risk factor for non-SLN metastases. This finding aids surgeons and oncologists in devising a plan for local axillary treatment, preventing both over- and undertreatment.

## Introduction

According to the International Agency for Research on Cancer’s GLOBOCAN predictions of cancer incidence and death, as of 2020, there may be 2.3 million new instances of breast cancer globally (or 11.7% of all cases), overtaking lung cancer as the most frequent disease among women [1]. Breast cancer accounts for 24.5% of new cases and 15.5% of deaths in women globally, with figures of 19.9% and 9.9% in China, respectively [1]. The status of axillary lymph nodes is a crucial predictor of outcomes in early-stage breast cancer. Axillary lymph node dissection (ALND) has traditionally been the most effective method for assessing the extent of lymph node metastasis. However, ALND can lead to complications such as lymphedema, impaired arm mobility, and sensory disturbances. A 10-year follow-up revealed that ALND did not benefit patients with SLN micrometastases [2]. Sentinel lymph node biopsy (SLNB) offers an alternative to ALND, avoiding postoperative complications while maintaining diagnostic accuracy and prognostic information [3]. Currently, SLNB is the standard surgical technique for early-stage breast cancer patients with negative axillary lymph nodes. Research shows that the survival rates between the SLN + ALND group and the SLN group in patients with negative SLN are comparable [4].

Following the ACOSOG Z0011 study, ALND may be omitted for clinical T1-2 breast cancer patients with 1–2 positive SLNs, with radiotherapy planned after breast-conserving surgery [5]. Only 41% of patients with macrometastases SLNs are estimated to develop additional axillary involvement, and this proportion was only 18% in micrometastatic or isolated tumor cells SLNs [6], and the risk of non-SLN metastasis is approximately 28.6–34.9% [7]. Following the AMAROS study, there was no significant difference in 10-year axillary recurrence rate, Disease-free survival, or overall survival between axillary radiotherapy alone and ALND in clinical T1-2 breast cancer patients with 1–2 positive SLNs [8]. As of 2014, according to the American Society of Clinical Oncology (ASCO), patients with breast-conserving surgery and 1–2 SLN metastases who receive conventionally fractionated whole breast radiation are not recommended for ALND [9]. Since not all patients with positive SLNs will experience subsequent lymph node metastasis, many researchers have explored prediction models for non-SLN metastasis in such patients. Predictors of axillary lymph node metastasis include SLN metastasis size > 2 mm, extra-nodal involvement, and the proportion of sentinel lymph nodes affected [10]. Tumor multifocality, tumor size, and lymphovascular invasion have all been identified as separate predictors of axillary lymph node metastasis [11]. In addition, in patients with 1–2 positive SLNs, non-SLN metastasis is more common in the invasive lobular carcinoma group compared to invasive ductal carcinoma [12].

Studies have shown that tumor-associated lymphangiogenesis, angiogenesis, and lymphovascular invasion are prerequisites for tumor metastasis and are associated with lymph node metastasis [13]. The spread of cancer cells into blood vessels and lymphatic vessels is a critical early event in tumor metastasis. Lymphovascular invasion independently affects the prognosis of breast cancer patients [14].

While some studies have examined whether lymphovascular invasion is a separate risk factor for axillary lymph nodes, the results have been inconsistent. Furthermore, most studies are based on Western populations, and little research has focused on SLN-positive early-stage breast cancer patients. Therefore, our study aimed to evaluate the relationship between lymphovascular invasion and non-SLN metastases in Chinese breast cancer patients with SLN positivity.

## Material and methods

### Study population

**Fig. 1:**
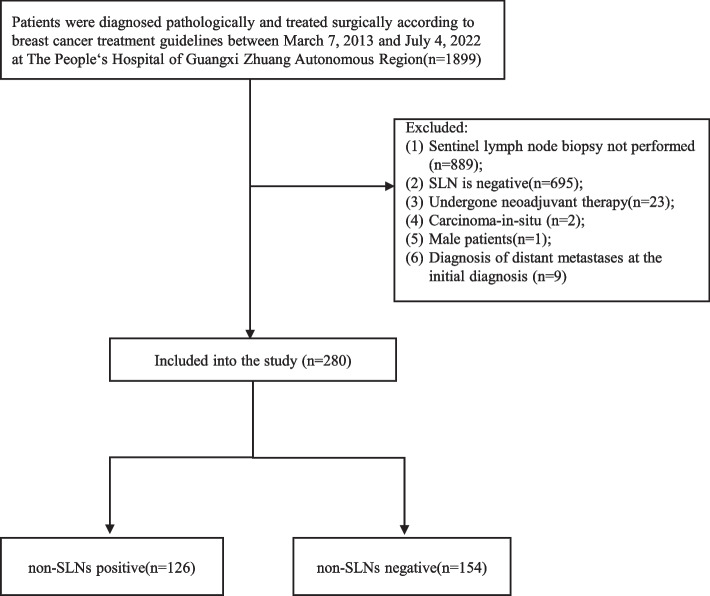
Flowchart for inclusion of patients from The People’s Hospital of Guangxi Zhuang Autonomous Region between March 7, 2013, to July 4, 2022

We conducted a retrospective analysis of patients with clinical T1-2 breast cancer who underwent surgical treatment in accordance with breast cancer treatment guidelines between March 7, 2013, and July 4, 2022, at The People’s Hospital of Guangxi Zhuang Autonomous Region. The following patients were excluded from the analysis: those who did not undergo sentinel lymph node biopsy (*n* = 889), those with negative sentinel lymph nodes (*n* = 695), those who underwent neoadjuvant therapy (*n* = 23), those with carcinoma in situ (*n* = 2), male patients (*n* = 1), and those diagnosed with distant metastases at the initial diagnosis (*n* = 9). A total of 280 individuals were eligible for participation in the study in the study (Fig. 1). The study protocol received approval from the Ethics Committee of the Guangxi Zhuang Autonomous Region People’s Hospital (Ethics-KY-QT-202205) and adhered to the ethical standards outlined in the World Medical Association Declaration of Helsinki. Due to the retrospective observational nature of the study, written informed consent was waived by the Ethics Committee of the Guangxi Zhuang Autonomous Region People’s Hospital.

### Clinical and laboratory data collection

We collected clinical data, including age, tumor size, menstrual status, estrogen receptor (ER), progesterone receptor (PR), human epidermal growth factor receptor-2 (HER-2), Ki-67, histology grading, lymph node metastasis, and lymphovascular invasion. The American Joint Committee on Cancer (AJCC) 8th edition staging system was employed for staging purposes.

### Diagnostic criteria

The American Joint Committee on Cancer (AJCC) 8th edition staging guidelines were used to diagnose and clinically stage breast cancer patients and assess lymph node status [15]. Tumor histology grading was determined using the Scarff-Bloom-Richardson grading system. Histological grading 3 was defined as the high group, and histological grading 1 and 2 were defined as the low group.

After employing methylene blue as a tracer during SLNB, suspected SLNs were removed, and intraoperative rapid frozen pathological examination was conducted. Formalin-fixed and paraffin-embedded tissue blocks were prepared after SLNB and ALND. These blocks were then serially sectioned to evaluate lymph node metastases. Frozen sections were stained with hematoxylin–eosin (HE), while formalin-fixed paraffin-embedded sections were stained with HE and subjected to immunohistochemistry (IHC). Two experienced pathologists examined the pathology. Ki-67 cutoff value was set at 20%, with ≥ 20% considered high and < 20% considered low [16]. For PR and ER, the cutoff value was established at 1%, with ≥ 1% being deemed positive and < 1% considered negative [17].

### Statistical methods

Numeric variables were presented as means with standard deviations or as medians with interquartile range (IQR), depending on the data's distribution. The Kolmogorov–Smirnov test was used to assess data normality, with a *p* value > 0.05 indicating a normal distribution. Categorical variables were expressed as frequencies and percentages. Comparison of continuous variables between two groups was performed using Student’s *t*-test, and categorical variables were analyzed with Pearson’s *χ*2 test. Multivariable logistic regression models were employed to examine the association between various clinicopathological variables and non-sentinel lymph node (non-SLN) metastasis. Unadjusted models were labeled as model 1, model 2 included adjustments for age and menopausal status, and model 3 further adjusted model 2 for the number of positive sentinel lymph nodes, Ki-67, HER-2, ER, and PR. We also conducted a stratified analysis based on age, ER, HER-2, histological stage, Ki-67, number of positive sentinel lymph nodes, PR, menstrual status, and tumor size. Statistical analysis was conducted using SPSS version 18 (IBM Corp., Armonk, NY, USA), and a *p* value < 0.05 was considered statistically significant.

## Results

### Characteristics of the subjects

Among the included individuals, 126 patients (45.0%) exhibited non-sentinel lymph node (SLN) metastases, while 154 patients (55%) who underwent ALND showed no non-SLN metastases. Table 1 presents the primary characteristics of the study population. Statistically significant differences were observed between the non-SLN negative group and the non-SLN positive group in terms of HER-2 status (negative: 82.5% vs. 69.8%; positive: 16.9% vs. 29.4%, *p* < 0.05), the number of positive SLNs (> 2: 11.04% vs. 35.71%, *p* < 0.05), tumor size (> 2 cm: 60.39% vs. 74.60%, *p* < 0.05), and lymphovascular invasion (32.47% vs. 74.60%, *p* < 0.05). In contrast, clinicopathological characteristics such as median age (51.72 ± 11.04 vs. 50.83 ± 10.44 years, *p* > 0.05), histological grading (high 10.4% vs. 7.1%; low 80.5% vs. 84.9%, *p* > 0.05), Ki-67 status (low 74.7% vs. 69.8%; high 24.0% vs. 30.2%, *p* > 0.05), PR status (negative 27.92% vs. 26.98%; positive 72.08% vs. 73.02%, *p* > 0.05), ER status (negative 16.88% vs. 16.67%; positive 83.12% vs. 83.33%, *p* > 0.05), and menopause (42.21% vs. 41.27%, *p* > 0.05) were not statistically significant.

**Table 1:** Main characteristics of the study population

Variable	Non-SLN negative (*n* = 154)	Non-SLN positive (*n* = 126)	*P* value
Age (years)	51.72 ± 11.04	50.83 ± 10.44	0.490
ER			0.962
Negative	26 (16.88%)	21 (16.67%)	
Positive	128 (83.12%)	105 (83.33%)	
HER-2			0.013
Negative	127 (82.5%)	88 (69.8%)	
Positive	26 (16.9%)	37 (29.4%)	
Unknown	1 (0.6%)	1(0.8%)	
Histology grading			0.325
High	16 (10.4%)	9 (7.1%)	
Low	124 (80.5%)	107 (84.9%)	
Unknown	14(9.1%)	10(9.1%)	
Ki-67			0.277
Low	115 (74.7%)	88 (69.8%)	
High	37 (24.0%)	38 (30.2%)	
Unknown	2(1.3)	0(%)	
Number of positive SLN			< 0.001
≤ 2	137 (88.96%)	81 (64.29%)	
> 2	17 (11.04%)	45 (35.71%)	
PR			0.861
Negative	43 (27.92%)	34 (26.98%)	
Positive	111 (72.08%)	92 (73.02%)	
Menopause			0.874
No	89 (57.79%)	74 (58.73%)	
Yes	65 (42.21%)	52 (41.27%)	
Tumor size			0.012
≤ 2 cm	61 (39.61%)	32 (25.40%)	
> 2 cm	93 (60.39%)	94 (74.60%)	
Lymphovascular invasion			< 0.001
No	104 (67.53%)	32 (25.40%)	
Yes	50 (32.47%)	94 (74.60%)	

### Association between lymphovascular invasion and non-sentinel nodal metastasis

The association between lymphovascular invasion and non-SLN metastasis is presented in Table 2. According to the results of multivariate logistic regression analysis, lymphovascular invasion, and non-SLN metastases exhibited a significant correlation (odds ratio [OR] = 6.11; 95% confidence interval [CI], 3.62–10.32) even after adjusting for age, menopause, number of positive SLNs, Ki-67, HER-2, ER, and PR.

**Table 2:** Association between lymphovascular invasion and non-SLN metastasis in patients with SLN-positive breast cancer

Model	*B*	Wald	OR	95% CI	*P*
Unadjusted model 1	1.810	45.814	6.11	3.62–10.32	0.000
Adjusted model 2	1.810	45.814	6.11	3.62–10.32	0.000
Adjusted model 3	1.774	40.303	5.894	3.41–10.19	0.000

*Model 1* univariable logistic regression model; *Model 2* including age, menopause; *Model 3* including model 2 covariates plus number of positive SLNs, Ki-67, HER-2, ER, PR

Table 3 displays the findings of the stratified analysis of the relationship between lymphovascular invasion and non-SLN metastasis. With the exception of participants with more than two positive SLNs, the connection between lymphovascular invasion and non-SLN metastasis in the stratified assessment aligned with the results of the multivariable logistic regression analysis.

**Table 3:** Association between lymphovascular invasion and non-SLNs metastasis according to baseline characteristics

Sub-group	Lymphovascular invasion		Non-lymphovascular invasion		OR (95%CI)	Mutually adjusted
	Total	Metastasis	Total	Metastasis	Crude	
ER						
Negative	28	16	19	5	3.7 (1.1, 13.2)	32.9 (2.4, 453.4)
Positive	116	78	117	27	6.8 (3.8, 12.2)	6.9 (3.6, 13.3)
HER-2						
Negative	105	66	110	22	6.8 (3.7, 12.5)	7.0 (3.5, 14.2)
Positive	38	28	25	9	5.0 (1.7, 14.8)	11.6 (2.4, 56.3)
Histology grading						
High	7	4	18	5	3.5 (0.6, 21.4)	inf. (0.0, Inf)
Low	132	85	99	22	6.3 (3.5, 11.5)	6.1 (3.2, 11.6)
Ki-67						
Low	94	61	109	27	5.6 (3.1, 10.3)	5.9 (2.9, 11.8)
High	50	33	25	5	7.8 (2.5, 24.3)	12.4 (3.1, 49.2)
Number of positive SLN						
> 2	42	34	20	11	3.5 (1.1, 11.2)	2.6 (0.6, 11.9)
≤ 2	102	60	116	21	6.5 (3.5, 12.0)	7.8 (3.9, 15.8)
PR						
Negative	44	27	33	7	5.9 (2.1, 16.6)	23.4 (4.2, 129.7)
Positive	100	67	103	25	6.3 (3.4, 11.7)	6.0 (3.0, 12.0)
Menopause						
No	88	56	75	18	5.5 (2.8, 11.0)	4.9 (2.3, 10.8)
Yes	56	38	61	14	7.1 (3.1, 16.1)	9.6 (3.5, 26.3)
Tumor size						
≤ 2 cm	36	20	57	12	4.7 (1.9, 11.7)	3.9 (1.4, 11.4)
> 2 cm	108	74	79	20	6.4 (3.4, 12.3)	8.9 (4.0, 19.4)
Age group						
≤ 50 years	65	13	78	54	9.00 (4.15, 19.53)	8.0 (3.2, 19.5)
> 50 years	71	19	66	40	4.21 (2.05, 8.66)	4.9 (2.1, 11.3)

The non-lymphovascular invasion group was the reference group. Each stratification adjusted for all the factors (age, ER, HER-2, histology grading, Ki-67, number of positive SLN, PR, menopause, and tumor size) except the stratification factor itself

## Discussion

The condition of the axillary lymph nodes significantly influences the treatment plan and prognosis for patients with primary breast cancer. SLNB has long been a safe and reliable method for evaluating axillary lymph node metastasis in clinically cN0 invasive breast cancer. If SLNB results confirm SLN metastasis, further ALND is performed. The findings from the IBCSG 23–01 and ACOSOG Z0011 trials suggest that ALND is not necessary for most patient populations with 1–2 SLN metastases who choose breast-conserving surgery and whole breast radiation therapy [4, 5]. More than 60 to 70% of patients with 1–2 SLN metastases do not require ALND or radiotherapy as regional treatment. In China, doctors often recommend ALND for patients with positive SLNs due to the low rate of breast-conserving surgery, unequal distribution of medical resources, and regional population differences [18]. However, patients prepared for breast-conserving surgery typically have smaller tumor sizes, hormone-receptor positivity, fewer positive SLNs, and an absence of lymphovascular invasion c. As our enrolled patients had no exclusion criteria related to breast surgery, the findings of this study apply to all surgical approaches. The results of the AMAROS study showed no difference in axillary recurrence rate, overall survival, and disease-free survival between ALND and axillary radiotherapy after a median follow-up of 10 years in cT1-2 breast cancer patients with 1–2 positive SLNs compared to each other [8]. However, considering the cost of treatment, adverse effects, and time cost, some cT1-2 breast cancer patients with 1–2 positive SLNs are not treated with radiotherapy as planned, especially in developing countries [19]. And more than 90% of cancer patients with radiotherapy would develop radiation dermatitis to a greater or lesser extent [20]. Can cT1-2 breast cancer patients with 1–2 positive SLNs without a radiotherapy plan be spared ALND, and what is their risk of axillary metastasis-related? Several previous studies found that 20% to 60% of patients were found to be free of non-SLN metastases after ALND. Therefore, these patients received unnecessary axillary therapy axillary therapy [21–23]. In the study by Meng, L. et al. 448 (62.7%) patients with 1–2 positive SLNs showed no non-sentinel lymph node metastases on postoperative pathological sections [24]. In our study, only 126 (45%) of the SLN-positive patients were confirmed to have non-SLN metastasis after ALND. This indicates that the other 154 patients (55.0%) underwent unnecessary ALND. Among patients with just 1–2 SLN metastases, 81 patients (37.2%) exhibited non-SLN metastasis. aligning with previous studies by Straver, M. E, Lei Meng, and Weiqi Gao [7].

Therefore, it is increasingly important to accurately predict non-SLN metastasis. Several studies have been conducted by scholars in the past, but their results vary. Andersson Y. et al. showed that tumor size and histological grade were significantly correlated with non-SLN status [25]. In the literature report by X Y Wang and Lei Meng, the histological stage was an independent prognostic factor for non-SLN metastatic disease [26]. However, these conclusions do not align with the findings reported by Amina Maimaitiaili [27]. Siem A. Dingemans's study demonstrated that in patients with SLN metastases, tumor size > 2 cm was a predictor of non-SLN metastasis [28]. In our study, the number of SLN metastases, tumor size, and HER-2 expression were associated with non-SLN metastasis, consistent with previous research [10]. However, the histological stage did not demonstrate predictive value for non-SLN metastasis. The small sample size or the presence of false-negative and false-positive SLNs may be responsible. The success rate of SLN biopsy using double-tracer methods, such as fluorescent dye, was higher than that of single-tracer methods [29].

A retrospective cohort study including 602 patients revealed that factors such as age, menopausal status, tumor size, histologic grading, hormone receptor status, HER-2 status, and lymphovascular invasion did not act as independent predictors for non-SLN metastasis [30]. In the investigation conducted by Lale, A et al., clinicopathological aspects, namely HER-2 positivity, perineural invasion, SLN extranodal extension status, and a metastatic SLN diameter exceeding 10.5 mm, were found to be distinct risk factors for patients with breast cancer non-SLN metastasis. In univariate analysis, a notable increase in patients with lymphatic invasion and vascular invasion was observed; however, these associations were found to be non-significant in the multivariate analysis [31]. Furthermore, the study suggested a close association between non-SLN metastases and lymphovascular invasion [32]. It has been shown that vasculogenic mimicry in solid tumors is associated with a higher lymph node metastasis group and a poorer Nottingham prognostic index. The occurrence of vasculogenic mimicry and phosphorylation of Ephrin type-A receptor 2 are independent prognostic factors in breast cancer and are associated with disease aggressiveness and progression [33]. A meta-analysis, including 624 patients, confirmed a correlation between primary tumor lymphovascular infiltration and the methods used for its detection with non-SLN metastases [34]. Lymphovascular invasion emerged as an independent prognostic factor in the context of breast cancer. In accordance with the tumor metastasis cascade theory, the infiltration of tumor cells into blood vessels and entry into the circulation represent the early pivotal events in tumor metastasis [14]. Several studies have underscored the status of lymphovascular/vascular invasion as an independent prognostic factor for axillary metastasis from lymph nodes. However, pathological detection, it can be influenced by differing methods of sampling, film preparation, staining, and interpretation. The reported prevalence of a positive rate for lymphovascular/vascular invasion ranges from 21.2 to 47% [35]. This variation has some influence on the predictive value of vascular invasion for non-SLN metastases. In our study, out of 144 individuals (51.4%), all confirmed by IHC had a lymphovascular invasion. Univariate logistic regression analysis revealed a significant association between lymphovascular invasion and non-SLN metastasis (odds ratio [OR] = 6.11; 95% confidence interval [CI], 3.62–10.32), even after adjusting for variables such as age, menopausal status, number of positive SLNs, KI-67, HER-2, ER, and PR. The correlation between lymphovascular invasion and non-SLN metastases remained significant after multivariate regression analysis. Both multivariable logistic regression analysis and stratified analysis confirmed a correlation between lymphovascular invasion and non-SLN metastases. Hence, lymphovascular invasion stands as an independent risk component for non-SLN metastases.

To summarize, non-SLN metastases are associated with the number of SLN metastases, tumor size, HER-2 expression, and lymphovascular invasion. Patients with HER-2 positive status, more than 2 positive SLNs, tumor size exceeding 1 cm, or lymphovascular invasion are at an increased risk of developing non-SLN metastases. Lymphovascular invasion emerges as an independent risk factor for non-SLN metastasis in invasive breast cancer patients with 1–2 positive SLNs. Lymphovascular invasion not only aids in surgical decision-making but also offers guidance for adjuvant treatment options. Patients with 1 or 2 SLNs testing positive but not receiving ALND or axillary radiotherapy face the risk of insufficient adjuvant treatment, particularly if they exhibit lymphovascular invasion, which raises the possibility of metastasis. In principle, the decision to administer adjuvant chemotherapy or radiotherapy can be informed by the presence or absence of vascular infiltration. In clinical practice, tailored strategies should be developed based on the clinicopathological characteristics of individual patients to prevent complications stemming from transitional axillary treatment. In cases involving lymphovascular invasion, the selection of axillary local treatment strategies should be made with great care to avoid inadequate treatment.

It is important to acknowledge some potential limitations of this study. Firstly, a single-tracer method was utilized for sentinel lymph node biopsy, potentially leading to false-negative or false-positive sentinel lymph nodes. In this study, methylene blue dye was employed for subareolar injection, which results in higher identification rates when methylene blue dye alone is used as a tracer [36]. However, subareolar injection complicates the visualization of internal mammary lymph nodes. Internal mammary lymph nodes, a significant metastatic route second only to axillary lymph nodes, serve as an independent prognostic factor for breast cancer [37]. Our study’s use of subareolar injections may have resulted in the omission of some internal mammary lymph node data. Moreover, the study primarily included patients with outer quadrant breast cancer, and patients with inner quadrant breast cancer were underrepresented. Consequently, these variables were not included in the study. The metastasis of internal mammary sentinel lymph nodes in patients with inner quadrant breast cancer could not be adequately assessed in this study. Our study divided the histological grading into high and low groups according to aggressiveness. It could not assess the difference between histological grading 1 and 2 adequately. Patient follow-up was not conducted, preventing a comparison of patient prognoses.

## Data Availability

The datasets generated in the present study are available from the corresponding author on reasonable request.

## Abbreviations

SLN: Sentinel lymph node
non-SLN: Non-sentinel lymph node
ALND: Axillary lymph node dissection
SLNB: Sentinel lymph node biopsy
ER: Estrogen receptor
PR: Progesterone receptor
HER-2: Human epidermal growth factor receptor-2
HE: Hematoxylin-eosin
IHC: Immunohistochemistry
ITC: Isolated tumor cells
CI: Confidence interval
OR: Odds ratio
ORs: Odds ratios

## References

[1] Sung H (2021). Global Cancer Statistics 2020: GLOBOCAN estimates of incidence and mortality worldwide for 36 cancers in 185 countries. CA Cancer J Clin.

[2] Dixon JM, Cartlidge CWJ (2020). Twenty-five years of change in the management of the axilla in breast cancer. Breast J.

[3] Veronesi U (2003). A randomized comparison of sentinel-node biopsy with routine axillary dissection in breast cancer. N Engl J Med.

[4] Krag DN (2010). Sentinel-lymph-node resection compared with conventional axillary-lymph-node dissection in clinically node-negative patients with breast cancer: overall survival findings from the NSABP B-32 randomised phase 3 trial. Lancet Oncol.

[5] Giuliano AE (2011). Axillary dissection vs no axillary dissection in women with invasive breast cancer and sentinel node metastasis: a randomized clinical trial. JAMA.

[6] Straver ME (2010). Sentinel node identification rate and nodal involvement in the EORTC 10981–22023 AMAROS trial. Ann Surg Oncol.

[7] Gao W (2020). Axillary lymph node and non-sentinel lymph node metastasis among the ACOSOG Z0011 eligible breast cancer patients with invasive ductal, invasive lobular, or other histological special types: a multi-institutional retrospective analysis. Breast Cancer Res Treat.

[8] Bartels SAL, Donker M, Poncet C, et al. Radiotherapy or Surgery of the Axilla After a Positive Sentinel Node in Breast Cancer: 10-Year Results of the Randomized Controlled EORTC 10981-22023 AMAROS Trial. J Clin Oncol. 2023;41(12):2159-65. 10.1200/JCO.22.01565. 10.1200/JCO.22.01565 36383926

[9] Lyman GH (2014). Sentinel lymph node biopsy for patients with early-stage breast cancer: American Society of Clinical Oncology clinical practice guideline update. J Clin Oncol.

[10] Callejo IP (2005). Predictors of positive axillary lymph nodes in breast cancer patients with metastatic sentinel lymph node. Clin Transl Oncol.

[11] Viale G (2005). Predicting the status of axillary sentinel lymph nodes in 4351 patients with invasive breast carcinoma treated in a single institution. Cancer.

[12] Zhang J (2021). Analysis of sentinel lymph node biopsy and non-sentinel lymph node metastasis in invasive ductal and invasive lobular breast cancer: a nationwide cross-sectional study (CSBrS-001). Ann Transl Med.

[13] Nathanson SD (2018). Breast cancer metastasis through the lympho-vascular system. Clin Exp Metastasis.

[14] Valastyan S, Weinberg RA (2011). Tumor metastasis: molecular insights and evolving paradigms. Cell.

[15] Amin MB (2017). The Eighth Edition AJCC Cancer Staging Manual: Continuing to build a bridge from a population-based to a more "personalized" approach to cancer staging. CA Cancer J Clin.

[16] Muftah AA (2017). Ki67 expression in invasive breast cancer: the use of tissue microarrays compared with whole tissue sections. Breast Cancer Res Treat.

[17] Sidhu B (2018). American Joint Committee on Cancer (AJCC) Cancer Staging Eighth Edition: Prognostic vs Anatomic Path Stage Group for Breast Cancer. J Registry Manag.

[18] Huang N-S (2016). Surgical management of breast cancer in China: A 15-year single-center retrospective study of 18,502 patients. Medicine.

[19] Gu L (2021). Comparing hypofractionated with conventional fractionated radiotherapy after breast-conserving surgery for early breast cancer: a meta-analysis of randomized controlled trials. Front Oncol.

[20] Singh M (2016). Radiodermatitis: a review of our current understanding. Am J Clin Dermatol.

[21] Chen K (2012). Validation and comparison of models to predict non-sentinel lymph node metastasis in breast cancer patients. Cancer Sci.

[22] Pal A (2008). A model for predicting non-sentinel lymph node metastatic disease when the sentinel lymph node is positive. Br J Surg.

[23] Chagpar AB (2006). Prediction of sentinel lymph node-only disease in women with invasive breast cancer. Am J Surg.

[24] Meng L (2021). Development of a prediction model based on LASSO regression to evaluate the risk of non-sentinel lymph node metastasis in Chinese breast cancer patients with 1–2 positive sentinel lymph nodes. Sci Rep.

[25] Andersson Y (2012). Prediction of non-sentinel lymph node status in breast cancer patients with sentinel lymph node metastases: evaluation of the tenon score. Breast Cancer.

[26] Wang XY (2019). Risk factors and a predictive nomogram for non-sentinel lymph node metastases in Chinese breast cancer patients with one or two sentinel lymph node macrometastases and mastectomy. Curr Oncol..

[27] Maimaitiaili A (2018). Analysis of factors related to non-sentinel lymph node metastasis in 296 sentinel lymph node-positive Chinese breast cancer patients. Cancer Biol Med.

[28] Wang X (2022). Sentinel lymph node positive rate predicts non-sentinel lymph node metastasis in breast cancer. J Surg Res.

[29] Kedrzycki MS (2021). Meta-analysis comparing fluorescence imaging with radioisotope and blue dye-guided sentinel node identification for breast cancer surgery. Ann Surg Oncol.

[30] Majid S, Ryden L, Manjer J (2019). Determinants for non-sentinel node metastases in primary invasive breast cancer: a population-based cohort study of 602 consecutive patients with sentinel node metastases. BMC Cancer.

[31] Lale A (2020). Predictors of non-sentinel lymph node metastasis in clinical early stage (cT1-2N0) breast cancer patients with 1–2 metastatic sentinel lymph nodes. Asian J Surg.

[32] Dihge L, Bendahl PO, Rydén L (2017). Nomograms for preoperative prediction of axillary nodal status in breast cancer. Br J Surg.

[33] Mitra D (2020). Phosphorylation of EphA2 receptor and vasculogenic mimicry is an indicator of poor prognosis in invasive carcinoma of the breast. Breast Cancer Res Treat.

[34] Nottegar A (2016). Extra-nodal extension of sentinel lymph node metastasis is a marker of poor prognosis in breast cancer patients: A systematic review and an exploratory meta-analysis. Eur J Surg Oncol.

[35] Zhang S (2017). High lymphatic vessel density and presence of lymphovascular invasion both predict poor prognosis in breast cancer. BMC Cancer.

[36] Li J (2018). Sentinel lymph node biopsy mapped with methylene blue dye alone in patients with breast cancer: A systematic review and meta-analysis. PLoS ONE.

[37] Lee SK (2013). The clinical meaning of intramammary lymph nodes. Oncology.

